# Equity, diversity, and inclusion in developmental neuroscience: Practical lessons from community-based participatory research

**DOI:** 10.3389/fnint.2022.1007249

**Published:** 2023-03-16

**Authors:** Shayna La Scala, Jordan L. Mullins, Rengin B. Firat, Emotional Learning Research Community Advisory Board, Kalina J. Michalska

**Affiliations:** ^1^Department of Sociology, University of California, Riverside, Riverside, CA, United States; ^2^Department of Psychology, University of California, Riverside, Riverside, CA, United States; ^3^Korn Ferry Institute, Los Angeles, CA, United States; ^4^Community Advisory Board Members, Anonymized for Confidentiality, Inland Empire, CA, United States

**Keywords:** community-based participatory research (CBPR), community advisory board (CAB), community-engaged research, developmental neuroscience, latino/a/x families

## Abstract

Exclusion of racialized minorities in neuroscience directly harms communities and potentially leads to biased prevention and intervention approaches. As magnetic resonance imaging (MRI) and other neuroscientific techniques offer progressive insights into the neurobiological underpinnings of mental health research agendas, it is incumbent on us as researchers to pay careful attention to issues of diversity and representation as they apply in neuroscience research. Discussions around these issues are based largely on scholarly expert opinion without actually involving the community under study. In contrast, community-engaged approaches, specifically Community-Based Participatory Research (CBPR), actively involve the population of interest in the research process and require collaboration and trust between community partners and researchers. This paper outlines a community-engaged neuroscience approach for the development of our developmental neuroscience study on mental health outcomes in preadolescent Latina youth. We focus on “positionality” (the multiple social positions researchers and the community members hold) and “reflexivity” (the ways these positions affect the research process) as conceptual tools from social sciences and humanities. We propose that integrating two unique tools: a positionality map and Community Advisory Board (CAB) into a CBPR framework can counter the biases in human neuroscience research by making often invisible–or taken-for-granted power dynamics visible and bolstering equitable participation of diverse communities in scientific research. We discuss the benefits and challenges of incorporating a CBPR method in neuroscience research with an illustrative example of a CAB from our lab, and highlight key generalizable considerations in research design, implementation, and dissemination that we hope are useful for scholars wishing to take similar approaches.

## Equity, diversity, and inclusion in developmental neuroscience: practical lessons from community-based participatory research

Mental health concerns account for a considerable percentage of the United States (U.S.) disease burden (Substance Abuse and Mental Health Services Administration, 2020) and are highly prevalent among ethnically and racially minoritized adults and youth (Dankwa-Mullan et al., 2010). As magnetic resonance imaging (MRI) and other neuroscientific techniques offer progressive insights into the neurobiological underpinnings of mental illness and become more incorporated into the mental health research agenda, it is incumbent on us as researchers that we pay careful attention to issues of diversity and representation as they apply in neuroscience. Problematically, many discussions around these issues are based on academic expert opinion without involving the community under study. In contrast, community-engaged approaches, such as Community-Based Participatory Research (CBPR), actively involve the population of interest in the research process and depend on collaboration between community partners and researchers (Mikesell et al., 2013). Community partners provide opportunities for open conversation about lived experiences that can guide efforts for promoting inclusion. This article outlines a community-engaged approach to neuroscience that intentionally includes community members in the research process. Our goal is to share our lab’s experiences with incorporating CBPR approaches into developmental neuroscience protocols so that researchers can assess the opportunities afforded by such approaches and consider including them in their own work. We use our efforts to incorporate CBPR methods into our ongoing study on mental health and neurodevelopmental outcomes in preadolescent Latina youth as an illustrative example and provide specific tools, materials, and guidelines for future neuroscience research that aims to incorporate a community-engaged agenda.

Many neuroscience studies focus either intentionally on race and ethnicity (i.e., racialized perceptions or cognition) or study local samples with shared cultural, ethnic, or racial experiences and backgrounds. However, despite the diversity of the populations we study, an intentional focus on representing diverse voices in our research is often neglected by neurosciences. In fact, most neuroimaging studies do not even report the racial and ethnic demographic composition of their samples (Goldfarb and Brown, 2022). Equitable science should be a leading motive for neuroscience as we navigate a racialized terrain that disproportionately excludes historically stigmatized and oppressed groups from research. If our inferences aim to reflect generalizable conclusions that benefit basic science and clinical goals, all groups should be included in the scientific process.

Three leading factors contribute to equity issues plaguing human neuroscience research: (1) lack of diversity in the neuroscience workforce leading to unacknowledged bias in scientific assumptions and scientific agendas that are often not aligned with the goals of the community under study; (2) lack of diversity in research samples and over-representation of Western and highly educated societies relative to the global population resulting in biases favoring white research participants (Henrich et al., 2010); and (3) insufficient transparency about participant demographics in neuroscience research prohibiting demographic group comparisons across samples. Addressing these issues requires neuroscientists to become more culturally competent if they intend to work with specific marginalized populations, focusing on sensitivity in research questions, hypothesis formation, and especially research methods (Henrich et al., 2010; Webb et al., 2022a). Because demographic factors, including sex, race, ethnicity, and socioeconomic status affect (either directly or through associated mediators) neural structure, function, and related behaviors, overlooking diversity has major implications for scientific reproducibility, generalizability, and the development of prevention and intervention efforts. We contend that a community-engaged approach can help address the unacknowledged bias and lack of diversity and inclusion in neuroscience research.

In the following sections, we first highlight the ways in which human neuroscience research has historically ignored the experiences of marginalized groups and led to biased knowledge generation in neuroimaging. Next, we describe features of interdisciplinary methods that may be adopted to actively counter these biases in neuroscientific research. Specifically, we describe “positionality” as a tool for acknowledging contextualized social positions of the researchers and the community they study, and CBPR from sociology and public health as mechanisms for community-engaged research. Within the CBPR framework, we detail how to build a Community Advisory Board (CAB, a group of community members that collaborates with and advises the researchers) as a practical tool for collaborating with the community in an effort to promote diversity, equity, and inclusion. We conclude by discussing the benefits and challenges of incorporating a CAB in neuroscientific community-engaged research and highlight key generalizable considerations in research design, implementation, and dissemination that we hope are useful for scholars wishing to take similar approaches.

## Bias in neuroscientific research

Marginalized communities, particularly those who have experienced historical oppression due to their race and ethnicity, have not only been disproportionately excluded from neuroscientific research but have also been actively harmed by intentional and unintentional biases (Webb et al., 2022a). Importantly, the conclusions drawn from such biased research find their way back to the communities under study, which further escalates systemic biases and mistrust against scientists. As neuroscientists, it has taken us far too long to realize that our research questions, hypotheses, and methods can introduce biases if we single-mindedly focus on our own (often prejudiced) assumptions. If we do not open communication channels and check in with our research participants, we will invariably continue to exacerbate the problem. In extant research, certain subsets of the population, including Black and brown people, have been too often viewed as not worthy of studying scientifically, or too “challenging” to recruit, leading to severe underrepresentation of marginalized groups in neuroscientific research.

Black, Latina, and other women of color, who are further marginalized by the interaction of gender and race, are particularly absent in neuroscience research (Spates, 2012; Gatzke-Kopp, 2016). For example, a systematic literature review reported that women and racial/ethnic minorities were underrepresented in functional magnetic resonance imaging (fMRI) studies of cardiovascular disease (Jones et al., 2020). This is especially problematic as certain ethnically and racially minoritized groups, like Black people, experience elevated rates of cardiovascular disease compared to their white counterparts (Loehr et al., 2008; Leigh et al., 2016). Similarly in the mental health domain, Latina girls, who are also underrepresented in neuroscientific research, demonstrate higher levels of untreated anxiety relative to their Black and white peers (McLaughlin et al., 2007). This exclusion of racialized minorities in neuroscience research directly harms communities and potentially encourages the development of biased prevention and intervention approaches.

Many electrophysiological devices that inform physical and mental health treatments were not designed to handle phenotype variability, contributing to a systemic exclusion of and erasure of data from people with darker skin and coarse or curly hair (e.g., Afro-Latino/a/x identifying) (Parker and Ricard, 2022; Webb et al., 2022a). Taking the technology used by our lab as an example, MRI uses a head coil that restricts big, afro-textured hair and sew-in hair extensions can have metal tracks that prevent an individual from entering an MRI bore (Thompson, 2009). The MRI machine itself also places great demands on participants, particularly children. Children must approach a very large, gray, loud, and strange machine, lie down on a table, and allow the experimenter to slowly glide them into the confined space of the scanner bore, where their head is restricted. Any of these things alone may induce worry, stress, and negative affect, and MRI procedures have been demonstrated to elicit feelings ranging from minor apprehension to severe panic, to increase cortisol levels, and to activate the sympathetic nervous system. The experienced stress during an fMRI experiment can potentially profoundly influence baseline neural activity; the perception of task stimuli, task engagement, and performance; as well as the physiology leading to functional activation patterns (Michalska et al., 2020). The degree to which the scanner environment influences MRI data varies with dispositional traits and demographic variables, which, depending on the study population and design, can lead to inaccurate interpretations of the resulting MRI data. For minoritized children, who have not previously been exposed to medical or research environments, experiencing such a novel and scary procedure might prove particularly daunting. In addition, MRI-induced negative affect will likely be amplified in children exposed to lifelong racialized stressors, including distrust of medical services, exhibiting signs of threat hypervigilance. Therefore, what neuroscientists might deem as “atypical” or “problematic” responses in minoritized youth, may instead be driven by chronic stress experiences that potentiate pre-scan anxiety.

Mounting evidence shows the effects of lived experiences on psychological processes (Torres et al., 2011; Berger and Sarnyai, 2015; Harnett et al., 2019; Mekawi et al., 2020; Bird et al., 2021; Fani et al., 2021, 2022; Webb et al., 2022b). As such, methodological tools can both be subject to bias against certain phenotypes shared by marginalized races and capture individual differences resulting from experiences that may co-vary with those phenotypes. For example, mental health symptoms that can arise from the experiences of racism, such as post-traumatic stress disorder, anxiety, hypervigilance, and anhedonia, may be reflected in psychophysiology data (Martínez et al., 2014; Harnett et al., 2019). Racial differences in the neural and behavioral responses to threats have been, at least in part, attributed to exposure to negative life experiences (Harnett et al., 2019), which occur at disproportionately higher rates in communities of color (Slopen et al., 2016) and may provide new insight into the mechanisms underlying racial disparities in mental health.

## Approaches to counter bias

**FIGURE 1:**
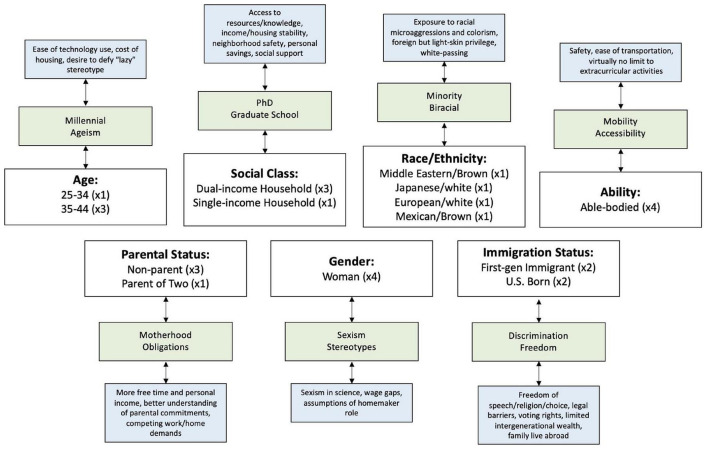
Positionality map. Tier 1 (white boxes) represents the identity categories selected for the current study (with a number representing how many individuals share a particular identity). Tier 2 (green boxes) specifies how these identities impact our lives and Tier 3 (blue boxes) elaborates further on the particularities of these identities.

Although no coding schema is perfect at encapsulating the rich and diverse identities of our research populations, researchers should be mindful and explicit of their selected operationalization of race and ethnicity. When testing group differences by gender, race, ethnicity, socioeconomic status, and their intersections, researchers should consider including measures developed from the perspective of the identified population, including measures that characterize larger systems of inequity and oppression. For example, measures quantifying experienced racism, life events, or neighborhood characteristics rather than (or in addition to) ethnic or racial categories can be incorporated to better identify why any observed differences exist and ascertain the structural systems that perpetuate them. This type of bottom-up thinking and operationalization in neuroscience has been termed “situated neuroscience” by feminist neuroscience scholars (Einstein, 2012; Walsh and Einstein, 2020), who argue that research findings must be contextualized within lived experiences. Whereas the traditional approach to science views the scientist in the role of the “observer” or the “outsider,” a *situated neuroscience* approach instead urges the scientist to consider their own social situation as well as that of the people they study. The multiplex of social locations (social group membership, geographical location, cultural background, age, etc.) from which the researcher sits in and their relative position with respect to others influences the way they experience their environment. Arguably, those at the top of social hierarchies can easily lose sight of the nature of social reality in their scientific pursuits and consequently miss critical questions about the social world (Harding, 2015). The practice of intentionally acknowledging how one’s social position shapes the generation of knowledge, or “positionality,” is a practice frequently employed by ethnographers (e.g., Rose, 1997; Reyes, 2020) and feminist theorists (Harding, 2004, 2015). We believe that positionality is not only useful for ethnographic and feminist research but also for neuroscience research (or in general all research). Through the practice of positionality and contextualizing the persons involved in research (including the researcher), we are able to “situate” our study and make the invisible relationships and taken-for-granted assumptions visible. One way to delineate positionality in a research study is by creating a so-called “positionality map,” sometimes also referred to as a “social identity map” or “standpoint map” (Jacobson and Mustafa, 2019). This map organizes in a diagram the standpoint of the scientist or knowledge-producer, making people more aware of the power inherent in positions of scientific authority. We elaborate on our own positionality mapping in forthcoming sections (see Figure 1 for our positionality map).

Whereas positionality involves explicitly identifying researchers’ social positions relative to the population under study, “reflexivity” is the process of critically examining how these positions affect the research process and resulting data (Chiseri-Strater, 1996). Reflexivity, alongside positionality, is an inductive approach favored by qualitative researchers and ethnographers conducting community-engaged field research. This approach aspires to reduce the power relations inherent in research and empower the community by facilitating their involvement in the design, implementation, and outcomes of research. Even though it might seem counterintuitive or even antithetical to combine such an inductive method with the often deductive objectives of neurosciences, emerging examples from a situated neuroscience approach indicate otherwise. For example, an influential community-engaged neurobiological study on the effects of female genital circumcision on the central nervous system and chronic pain in Somali Canadian women centered women’s experiences via a CAB that gave input and guidance on the study (Einstein, 2012). By reflecting on her own position as a white female immigrant American neuroscientist in Canada and co-producing knowledge using the definitions, standards, and perspectives of the community, Einstein (2012) portrayed the lived experiences of the community she studies alongside the neurophysiological data. This blended approach empowers both researchers and study participants as persons embedded in their environments and experiences and counters the frequently reductionist views of the brain. After all, isn’t the goal of neuroscience to reveal what experiences are “like” for people (i.e., neurobiological mechanisms for thinking, affect, behavior, development, etc.)? Below we outline our own approach to CBPR via the formation of a CAB in our neurodevelopmental study of Latina girls and their families.

## Community-engaged research

*A* “*community”* is an interdependent group of people that share sets of characteristics, culture, values, and norms and come together by a sense of overall care for what happens to one another, understanding that what happens to one individual affects many others as they navigate similar relationships within a social structure and specific geographical location (Nutbeam and Kickbusch, 1998; MacQueen et al., 2001). As researchers, we recruit from communities and study people who belong to communities, if not the whole communities themselves. Importantly, even though all human research is in fact the study of people embedded in communities, limited work considers the impact of this research *on* the community. For instance, qualitative social sciences traditionally have an interest in the communities they study and emphasize building relationships with the people who are studied, taking into account the community members’ perspectives and researchers’ own positionality (Jacobson and Mustafa, 2019). This approach is particularly useful when the research is focused on “hard to reach” populations who are socially excluded from sectors in society, which limits their access to resources and inhibits their motivation to seek resources (Flanagan and Hancock, 2010). Individuals with mental health disorders or children with anxiety or conduct problems can be considered hard-to-reach populations. Due to numerous and compounding structural barriers, people who belong to marginalized communities are often reluctant to participate in research, limiting generalizability of research findings on mental health outcomes.

In response to escalating demand at multiple societal levels (e.g., community leaders, policymakers, funding agencies) calling for the broadening of methodological approaches and involvement of impacted communities in research, we are experiencing a gradual (albeit slow) shift in public health research agendas (Ahmed and Palermo, 2010). *Community-engaged research*, conceptualized as an avenue through which the complex cultural issues that affect health disparities in underserved communities can be addressed (Michener et al., 2012), is defined as the process of working collaboratively with groups of people affiliated by: (a) geographic proximity, (b) special interest, or (c) similar situations, to address issues affecting the wellbeing of those people and encourages community-academic partnerships (Drahota et al., 2016). One of the most important aspects of community-engaged research is the condition that community members work with researchers as equal interested parties and actively shape the research they are a part of Andrews et al. (2013). There are several types of community-engaged research aimed at empowering the community under study (Annett and Rifkin, 1995; Fetterman et al., 1996; Rifkin, 1996; United States Environmental Protection Agency, 2015). In this article, we focus on CBPR, which takes a partnership approach to research by involving interested community partners in all facets of the research process (Cornwall and Jewkes, 1995; Faridi et al., 2007). In other words, CBPR asks: how will the lives of people in communities be impacted by a specific piece of research and do those people have a voice in whether and how the research will be conducted?

CBPR dates to the early 1930s and is predominantly used by public health researchers in generating health-enhancing programs that evolve through community members (Lewin, 1947; Faridi et al., 2007; Freire, 2018). This framework is embedded within public health education, but has become increasingly interdisciplinary, entering departments of sociology, psychology, and more (Medicine, 1997; Sun et al., 2022). As noted above, CBPR is a collaborative research approach that actively involves communities directly affected by the issue under study in every aspect of the research process, from design to dissemination. This brings about mutual ownership of the products produced from research (Viswanathan et al., 2004). CBPR aims to improve health and well-being via a reciprocal transfer of expertise between the research team and community partners with an overarching goal of bi-directional learning, equal exchange of knowledge, and shared power in decision making (Castille, 2018). CBPR initiatives such as CABs include community members as research partners at multiple steps in the research process. This relationship offers researchers the opportunity to situate themselves and the people they study and provides a baseline for reflexivity. The researcher learns about and acknowledges shared and distinct experiences and social locations of persons involved in the study.

CBPR is employed by first identifying a key population or geographic location of interest, which takes place in the planning stages of research. As is the case for our work, this population might be connected to the local sample of a research study. CBPR is one of the most intensive community-engaged research approaches, in which researchers and community members share power in the identification of research topics and questions, the application of results, and the dissemination of findings (Minkler, 2010; Yuan et al., 2016). Of note, partnerships systematically embedded in the research process maximize the applicability of the research findings (Pasick et al., 2010). Overall, CBPR aims to enhance the interpretation of an issue via collaboration with those most affected and subsequently integrate that knowledge for the improvement and wellbeing of the community of focus (Green et al., 1995; Israel et al., 2001).

All phases of a CBPR project involve a close-knit collaboration and a strong foundation for mutual understanding, respect, and trust between the participating members. Community collaborators act as informational liaisons between scientists and community, typically forming a group of approximately 6–12 people. These individuals can be interested parties in the community, members of the community themselves, or under-represented individuals. They make up the CAB and agree to this position with full awareness of what participation in this capacity entails. This means that there must be mutually agreed upon goals and a co-generated governance structure, including rules of conduct, ensuring continued collaboration from the beginning to the end stages of a research project. In the following section, we provide a concrete example of a neuroscience study conducted by our research team that integrates a CBPR research strategy.

## An illustration from a community-engaged neuroscience approach

The Kids Interaction and Neurodevelopment (KIND) Laboratory at the University of California Riverside (UCR) leverages MRI and psychophysiological methods to study the neurodevelopment of emotion understanding in typically developing children as well as children with pediatric anxiety and disruptive behavior problems. The primary ongoing longitudinal study at the KIND Lab, the *KIND Lab Girls Study* (*KLG Study*), focuses on preadolescent Latina girls and their families. Around the end of 2019, shortly before the start of the COVID-19 pandemic, the KIND Lab began a collaboration with colleagues from the UCR sociology department (SLS and RBF) to center the voices of the local community (Latino/a/x families in the Riverside catchment area) and better understand the cultural experiences shaping their mental health.

UCR is situated in the two-county area in Southern California, referred to as the Inland Empire (IE). This rural area encompasses the largest county in the United States, San Bernardino county, characterized as a major warehousing and distribution hub for global corporations (Ebner, 2020). Although immigration and emigration have undergone transitions in recent years, the IE remains home to a large population of Latino/a/xs experiencing elevated levels of psychological distress that raises mental health concerns (Barragán et al., 2020). This population also significantly under-utilizes mental health services compared to non-Hispanic white Americans, making them a priority at-risk community (Rao et al., 2007). Underutilization of services potentially stems from experiences of stigma and discrimination (Link and Hatzenbuehler, 2016). Although there has been an overall shift in mental health services, cultural stigmas persist and serve as barriers to attitudes toward help-seeking (Vogel et al., 2006; Wei et al., 2015; Zhou et al., 2022). These barriers around mental health stigma also affect the participation of these historically underserved communities in scientific research studies on mental health, particularly in adolescents.

The *KLG Study* did not start out with a CBPR emphasis, instead, it began with a focus on the neural bases of disruptive behavior disorder and conduct problems in Latina youth based on our prior work (Michalska et al., 2015, 2016). However, in the process of collecting data and informally speaking with families, we learned that what girls in our community were instead struggling with was elevated panic, and separation and social anxiety, exacerbated by social stressors. Problematically, Latina adolescents experience more internalizing symptoms and higher rates of untreated anxiety than their white, Black, and Latino peers (McLaughlin et al., 2007; Tran et al., 2014; Kann et al., 2018; Stafford and Draucker, 2020). Indeed, to date, even though we did not specifically recruit *KLG Study* participants for anxiety symptoms, 28.3% of child participants meet diagnostic criteria for anxiety disorder based on parental reports on the Screen for Child Anxiety Related Emotional Disorders (SCARED; Birmaher et al., 1997), and 30.8% have levels of anxiety in the subclinical range. In our interviews, we were also struck by the unique socialization experiences of Latinx communities that potentially impact mental health outcomes. For example, Latino/a/x parents play a pivotal role in shaping how their children process emotions (Michalska and Davis, 2019) and understand experiences that relate to ethnic-racial discrimination (Ayón, 2016), which may play a protective role in the association between racialized stressors and children’s mental health symptoms. Parents influence the development of behavioral adjustments to help or hinder their children’s emotion regulation in emotionally charged encounters (Kochanska, 2002). Thus, our research pays particular attention to the influence of ethnic-racial value socialization practices among Latina mothers on their children’s emotion expression, recognition, and regulation when they engage in threat and safety learning, as well as mother-child interactions during tasks. Based on this context and with guidance from the UCR Center for Health Disparities Research, we re-evaluated the aims of our study to prioritize families’ concerns on our research agenda and center their lived experiences. We began by creating our own positionality map as a platform for self-reflexive analysis (see Figure 1).

### Researcher positionality mapping

As reviewed above, a positionality map allows us to critically examine our research roles as they pertain to identity, power, and privilege, and develop attitudes that embrace cultural humility (Collins et al., 2018). Because researchers are primary vessels through which information is filtered to generate data, our social identities affect how we interpret this information (Leibing and McLean, 2007; McLean, 2007; Day, 2012; Jacobson and Mustafa, 2019). Identities can include race/ethnicity, sexual orientation, ability, age, and citizenship (Dhamoon and Hankivsky, 2011; Collins, 2015; Jacobson and Mustafa, 2019). Because identities are fluid and ever-changing, identifying them is a complex process, particularly in the context of developmental research where participants are followed longitudinally over extended periods of time. Here (and in general), we believe that positionality mapping should be a routine and regular practice. Our positionality map represents an initial step in reflecting on how our current identities shape our perspectives as researchers (Day, 2012; Jacobson and Mustafa, 2019) and we intend to return to it regularly as the *KLG Study* progresses. We have shared our positionality maps with select CAB members during a workshop series and we intend to incorporate them in future meetings with the entire CAB.

Informed by previous work on positionality (Jacobson and Mustafa, 2019), our research team reflected on the identities that were most relevant for the focus of the *KLG Study* on Latina girls and their families. Per prior guidelines, we focused on facets of our social identity that help us better understand the power relations imbued in our research, as well as those uniquely impacted by the social and political climate our KIND Lab is located in. Tier 1 (white boxes) represents the following selected social identities: social class, race/ethnicity, ability, parental status, gender, age range, and immigration status. The research team that completed the positionality mapping exercise included two faculty and two graduate students (in addition to a larger research group comprised of graduate and undergraduate trainees in the KIND Lab) who participated in CAB meetings. The two faculty members included: (1) a white Polish-Austrian woman with expertise in developmental neuroscience and pediatric anxiety; (2) a Brown Turkish woman with expertise in inter-group relations and racial health disparities. The two graduate students included: (1) a Latina mother and doctoral student in sociology with training in school and medical sociology, who had also completed coursework in community-engaged research, and (2) an Asian-American woman and doctoral student in developmental psychology with training in the neurodevelopment of anxiety in underrepresented youth and their parents. Tier 2 (green boxes) specifies how each of these identities impact our lived experiences and Tier 3 (blue boxes) further elaborates on the nuances of these identities. As an example, we discussed our team’s parental status and ethnic composition due to our focus on maternal parenting among Latina mothers. One of our four research team members is also a Latina mother, making them uniquely equipped to empathize with community members’ parental demands, whereas other team members acknowledge they have more free time and personal income or may benefit from racial privilege due to European ancestry. Mapping identities can shed light on our explicit and hidden identities, which can strengthen a study (i.e., shared gender identities among the research team and our participants in our study) or reveal our hidden assumptions and worldviews (i.e., racial privilege, social class advantages, and ableism). The positionality map fosters awareness of our positions and the way they shape the production and interpretation of knowledge (Campbell and Wasco, 2000; Jacobson and Mustafa, 2019). For example, it has allowed our research team to better identify our “blind spots” and expand our group to include and recruit members whose identities were previously inadequately represented. It has also created an opening for eliciting counternarratives that deprivilege researcher expertise and enable us to ask questions that were initially not on our research agenda but the community considers urgent. Doing so as a collaborative process allows for multiple interpretations from a variety of entry points and perspectives. Our plan is to sustain an awareness of these multiple perspectives as we continue to collect and analyze our data.

### Community-researcher partnership

With guidance from the UCR Center for Health Disparities Research, the research team partnered with Latina mothers residing in the IE and participating in the *KLG Study* to form the project’s CAB, named the “Emotional Learning Research Community Advisory Board”. Following prior recommendations (Newman et al., 2011) our research team targeted 10–15 community advisors. All CAB-related procedures were approved by the Institutional Review Board at UCR. In our submission for protocol approval, we described the CAB meetings as a series of community outreach discussion meetings that would be conducted with a subset of our participants who had consented to be re-contacted by the lab. We specified that these meetings would take the form of an informal discussion in a neutral location outside the institution (e.g., a local library) to make everyone feel comfortable in the discussion space. We note that due to the COVID-19 pandemic, these meetings were ultimately carried out in a virtual format via Zoom. Aligning with CBPR research orientation, meetings provided a space for CAB members to give feedback on our research efforts, specifically, as well as how we might be of service to the community more generally. Even though participation was voluntary, we thought it important for participants to be compensated for their time, that we would not engage in formal data collection, and that meetings would not be used for data collection purposes, but rather an evaluation of the laboratory’s current functions and operations.

Informed by previously established criteria (Newman et al., 2011) as well as our continued working relationships with our participating families, we identified specific community members of diverse socioeconomic backgrounds and age groups who might serve as community representatives. These representatives included mothers who had participated in previous studies conducted by our lab, and whose participating children ranged in anxiety symptoms from non-anxious to clinically anxious thresholds. We contacted these individuals via phone and email, and those who expressed interest were sent a virtual consent form (see Supplementary material) in which they agreed to attend two 60–90-min virtual Zoom meetings, scheduled approximately 6 months apart (June 2020 and December 2020), with subsequent meetings planned. We had a 100% retention rate across the two meetings, with the same eight mothers who attended the first CAB meeting also attending the second. Meeting dates and times were determined based on the general availability of the CAB members who were compensated at a rate equal to what the lab pays traditional research participants per meeting ($50–$100). We took creative measures during COVID stay-at-home rules, and to express our appreciation for their time and effort, our research team had pizzas delivered to each representative’s home address (with their consent) in the hour prior to the first meeting. Each of the two meetings are explained in detail in the proceeding section, drawing from our meeting agenda script (see Supplementary material for an outline of the first meeting).

## Community advisory board meetings

CAB meetings were led by the first author and attended by all three co-authors, as well as other KIND Lab trainees. Meetings were scheduled for 1 hour. Prior to each meeting, the first author created an agenda that was distributed to the CAB members along with materials to be discussed. The agenda tentatively included topics in a specific order, with the first meeting focused on our laboratory recruitment efforts (e.g., traction of our recruitment fliers) and research protocols (e.g., comfort with fMRI) and the second focused on members’ experiences of sociodemographic determinants of mental health (e.g., race/ethnicity, political ideology). It was agreed that if time ran out we would roll topics over to the next meeting.

Our priority was to build rapport between our research team and the community representatives (Alvarez et al., 2006). For Latino/a/x families, structural barriers such as lack of transportation, need for childcare, costs of participation related to lost time at work, competing family responsibilities, and limited language-appropriate recruitment and informed consent processes can all engender anxiety and mistrust of the scientific community. One way the team established trust was via a CAB facilitator, the first author, who identifies as a Latina mother and who led the CAB meetings. We began the meeting with a light icebreaker asking people to share their names and favorite sandwich. After introductions and the icebreaker, questions from the agenda were guided by a facilitator and the co-principal investigators (KJM and RBF) (see Supplementary material). The first set of questions asked about the clarity of our *KLG Study* consent forms. CAB members shared that there was sufficient information and they appreciated the straightforward language. They also noted that they felt comfortable asking questions if anything was unclear. Building rapport had a positive impact on outcomes for researchers and community members simultaneously, and some research suggests that rapport holds unique promise for community transformation as it involves community members themselves, in contrast with traditional research retrieval methods (Sousa, 2022).

The next set of questions centered around any worries and anxieties about CAB members’ overall research experience in the *KLG Study*, specifically regarding clinical interviews and MRI scanning. To elicit constructive feedback on our protocols, we primed CAB members with neutral rather than valenced questions. Our aim was to establish a neutral starting point for the conversation, and allow members to guide the direction of the discussion based on their personal experiences with our procedures. Most mothers shared how they and their daughters had positive interactions with KIND Lab researchers, and some mothers revealed that their daughters were initially anxious and overwhelmed in anticipation of the MRI, due to their unfamiliarity with the equipment.

Next, community members reflected on the ongoing experiences of Latina girls in the community and their motivation to participate in the *KLG Study*. One member described the dearth of psychological services, asserting that youth were disadvantaged by the lack of investment in mental health practitioners in the IE region. Another member articulated their motivation to elevate the perspectives of women of color to science. They discussed the impact of generational experiences and cycles of trauma they were trying to break and their desire to expose their daughters to institutions of higher education and scientific inquiry centered around mental health and wellbeing. Some mothers disclosed that one of the factors motivating them to participate in the study was the opportunity to show their daughter what a university campus looks like.

Another set of questions covered the recruitment process. Our research team asked about members’ motivations for participating in the *KLG Study*. We shared our current recruitment flier and solicited feedback from CAB members. Among other observations, CAB members pointed out that the language did not reflect the community values or the way they thought about their children’s behaviors, concluding that the flier seemed targeted toward college students, rather than families. All members noted that the flier did not appear child-friendly or family oriented, adding it did little to capture their attention. They then brainstormed ways to modify the flier so that it could speak to the specific needs of children in their community, recommending more colors, different wording, and additional information about resources and payment. Ambiguity was generally viewed as a deterrent to potential participation. CAB members also shared recommendations on possible recruitment venues, and thoughts about engaging families in different spaces. Among the recommendations were guest speaking at schools, university tours of the lab to demystify the research and environment, and joining forces with other health networks in the IE. Engaging the community and offering training for members of the community align with one of the guiding principles of community-engaged research (Battaglia et al., 2020).

CAB members were invited to suggest how the KIND Lab could better serve their families, to which they responded by requesting resources that might act as a gateway to services like counseling and therapy. Members viewed their participation in our research as an entry point for conversations with their children about mental health. They also proposed ways we might earn the trust of community members who were more hesitant to participate in our research (e.g., via partnering with community health centers). These conversations sparked a discussion about what we as researchers of socioemotional development might be able to provide participating families, given our available resources. One CAB member expressed interest in art therapy, and several other members chimed in with enthusiasm agreeing that their children could benefit from such an approach. Thanks to this suggestion, the KIND Lab decided to design and implement an art therapy workshop series. Three Saturday morning workshops were hosted on campus, each lasting approximately an hour and a half, scheduled approximately 2 weeks apart. The goals of the workshops were to connect with community members and provide children with tools they could use to manage stressors through artistic expression.

**FIGURE 2:**
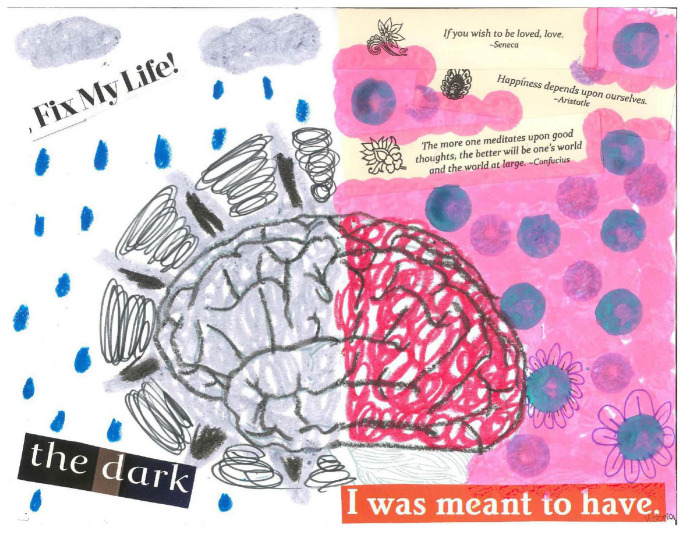
Art created by one of the participants in the art therapy workshop series.

The first art therapy workshop was facilitated by a professional art therapist, who invited participants to visually explore their emotions using paper, color markers, gel pens, glue, scissors, and magazines. The therapist guided children in exercises that prompted them to identify and challenge unhelpful thoughts, and use art as a coping strategy to regulate and overcome these thoughts when they arise (see Figure 2 for an illustrative example). Participants completed minor assent forms and parents completed consent forms, indicating permission for photographs to be taken by a professional photographer while they participated in the lessons. The second art workshop was facilitated by a professional dance artist, who led children through a series of movement and dance exercises that provided them with non-verbal, embodied tools for coping with anxiety and other challenging emotions. This workshop was held in a university theater and dance space that enabled ample freedom of motion. The third and final workshop of the year was facilitated by a self-published children’s author, who was also a CAB member. She shared how her journey of writing a book allowed her to cope with a difficult life circumstance and then led children in conceptualizing and creating their own storybooks using illustrations and narrative. Attendance at each workshop ranged from two children and their caregivers to ten children and their caregivers. At the end of the series, the team compiled participants’ artwork and workshop photos into customized printed books for each participant. Even though each child received a book to take home, all three workshops emphasized the healing quality of the creative process itself. It was thanks to our CAB members that we were able to provide a resource the community would find valuable.

The theme of the second CAB meeting centered around race, ethnicity, identity, and how to appropriately address participants, as well as the impact of ethnic-racial discrimination on children’s daily lives. The research team asked CAB members about their ethnic-racial identity and their preferred terms (e.g., Latinx vs. Latina/o). This generated lively discussion, with responses reflecting varied use of identity labels, with some CAB members expressing misgiving about the term “Latinx” as they are not of Latin descent while acknowledging differences in adoption depending on generation, geography, and even political orientation. One member disliked the term “Latinx” saying it felt too scholarly and did not accurately represent her identity as a Mexican-origin Latina woman. Others agreed that they did not identify with this term, adding that it sounded like an English-based construct and unnatural in Spanish. Based on this feedback and to center the voices of community members, our research team decided to adopt the community’s preferred ethnic identity labels for any communication with parents. However, in recognition that gender is not binary, we have also added the Latino/a/x in the current manuscript. A planned youth panel will similarly ask youth about their attitudes and preferences.

The second topic focused on political climate and immigration experiences. Because of the U.S. immigration policy context and surrounding anti-immigrant sentiment at the time of the second meeting (i.e., the Trump presidency), our research team asked about members’ attitudes toward disclosing immigration and discrimination experiences in a laboratory setting. Mothers generally felt that discussing immigration status might hinder participation due to fear of questions about legality, although some mothers shared that they would be willing to disclose their status if their identity were protected. Several CAB members also discussed the stigma of being a Latina immigrant in this country and the toll it can take on the mental health of the community. Comments in the meeting focused on how to incorporate these experiences into the team’s research agenda and regional mental health services more broadly, while protecting anonymity. U.S. immigration policy has grown more restrictive in recent decades subjecting Latino/a/x immigrant families to inequitable treatment on the basis of their actual or perceived immigrant status. Restrictive policies directed toward immigrants who are undocumented have untoward effects on the health of Latino/a/xs, regardless of their status. Being an immigrant and being undocumented have become conflated with being Latino/a/x; more specifically, assumptions about a person’s origin and legal status are based on racial markers (Ayón and Philbin, 2017). The *KLG Study* will be among the first to leverage neuroimaging to examine how associations between immigration threat and mental health shape brain development during transitions to adolescence.

The final theme of the meeting captured the notion that political ideology also influences emotional wellbeing, particularly given the tumultuous political divide during the time of the meeting. Mothers hinted at the toll the political climate was having on them and their daughters. We closed our meeting with a conversation about how the political atmosphere may interfere with people’s trust in science and subsequently impact participation rates. In response to the community members’ emphasis on the primacy of sociocultural context in shaping emotional health in their families, our research team substantially expanded existing KIND Lab protocols. For example, our team partnered with Dr. Cecilia Ayón, a faculty member in the Department of Public Policy with specific expertise in community-based research with Latino/a/x immigrant families at the intersection of sociopolitical context, immigrant health, family wellbeing, and ethnic-racial socialization. Dr. Ayón has engaged in research aiming to inform and assess the effectiveness of culturally and contextually grounded interventions. Guided by CAB member feedback and in consultation with Dr. Ayón, *KLG Study* data collection was modified to adapt a Latino/a/x parental ethnic-racial socialization questionnaire to a child-appropriate version that would be administered to children, among several additional questionnaires probing discrimination and socialization experiences.

**FIGURE 3:**
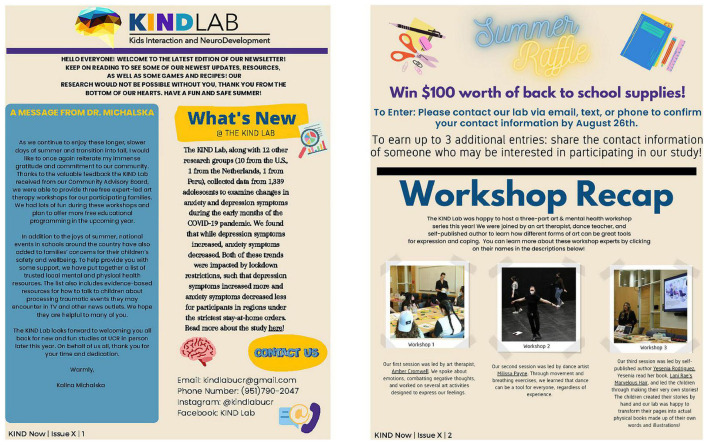
Excerpted pages from our KIND Lab Newsletter.

The *KLG Study* data analysis has been significantly impacted by the CAB meetings, with our biases and positionality informing the lens through which results are interpreted, disseminated, and communicated. Had our research team not incorporated a CBPR approach, many of our ongoing research directions would not exist. Beyond data and with community needs in mind, we share a biannual KIND Lab Newsletter with participating families (Figure 3), where we share any papers or conference proceedings that have been published with our sample, highlighting results in easily accessible language. We also keep a running tab of free or low-cost community mental and physical health resources that we update regularly.

In the following section, we want to leave other scientists in the neuroscience community with some considerations for building their own CAB and embarking on inclusivity within the scope of their work. Table 1 outlines the steps of consideration in developing the CAB and the purpose as a brief explanation. Labs may also want to consider the following:

**TABLE 1:** CAB considerations and purpose.

Consideration	Purpose
Schedule a team meeting to discuss topic options and logistics.	Ensure the team is on the same page of identifying 6–12 potential CAB members to meet with and topics to focus on.
Discuss positionality at the team meeting.	Become aware of team’s working identities and privileges that may affect the research by completing a positionality map individually and as a group.
Reach out to potential CAB members via whatever communication is appropriate and accessible (e.g., email, telephone, written mail, social media).	Begin rapport and provide information on what CAB participation entails.
Formalize CAB member participation with consent and agreement.	Safeguards transparency, accountability, and follow-through.
Secure compensation for CAB member participation.	Ensure the compensation amount for the CAB members’ time and effort is fair.
Schedule a team meeting to finalize the CAB meeting agenda, draft conversational questions for the CAB members, and elect meeting note takers and facilitators.	Establish clear roles for each member of the research team and have a list of questions/topics prepared for the CAB members (e.g., what are current issues in your community? what types of questions should be considered in the research?) This will ensure the meeting time is spent as efficiently as possible.
Distribute meeting information to CAB members.	Give CAB members a chance to reflect and prepare for the topics your team is planning to discuss.
Hold the CAB meeting.	Ensure the meeting works with your CAB members’ schedules and is considerate of their time. Progress through the planned questions and topics in a manner that is sensitive to the natural flow of the conversation (i.e., CAB members may prefer to spend more time on some topics than others). Allow CAB members to speak freely and only interject to break moments of silence or shift away from topics that have run their course.
Hold a brief team meeting following the CAB discussion.	While the meeting and feelings about the meeting are still fresh, briefly discuss what went smoothly and what needs adjusting in future CAB meetings.
Send thank you notes to CAB members and share meeting notes.	Build and maintain as much transparency with the CAB members as possible. This is foundational to CBPR.
Repeat steps to discuss the last meeting and plan for the next meeting.	Each meeting should inform the topics to be covered and adjustments to be made in the following meeting.
Determine how the CAB meeting information will be incorporated into the research and impact the community you are collaborating with.	Ensure information obtained during the CAB meeting is implemented into your research in concrete ways (e.g., consider topics the community has deemed especially important, offer resources the community would find the most helpful, etc.).

**Before the CAB meeting/considering population of interest:**

• Who is a part of the community you are considering a collaboration with?
• Who are the interested parties in the community?
• Who in your research team will take the lead to communicate with community members?
• What are the community members’ positionalities?
• What is your research team’s positionality?
• How will you recruit community members to become CAB members?
• What types of topics will be discussed at the CAB meeting?
• When will agreements, consent, and agendas be shared with CAB members?

**During the CAB meeting:**

• Where will the meeting take place to ensure equal grounds for CAB members and researchers?
• How will CAB members be compensated for their time?
• How will conversation during the meeting be navigated?
• How many team members will be present during the meeting?
• Who will lead the meeting? Who will take notes during the meeting?
• How will rapport be built with CAB members?
• How many and how often will meetings be?

**After the CAB meeting:**

• When, if any are planned, will the next meeting be?
• How will CAB members be retained?
• When will previous meeting notes be shared with CAB members?
• How will the information and knowledge shared in the meeting be of use to the community?

## Conclusion and implications

In this paper, we briefly highlighted the ways in which human neuroscience research has overlooked historically marginalized groups, reproducing systemic inequities. We propose that these biases can be revealed through practices and tools from interdisciplinary and qualitative research, such as positionality and reflexivity, and actively countered through a community-engaged research approach, particularly CBPR. In a pursuit to offer a detailed roadmap for integrating CBPR into neuroscience research, we outline the ways our lab has incorporated two specific CBPR methodologies, a positionality map and a CAB, into an ongoing, longitudinal neuroimaging study on the mental health outcomes of preadolescent Latina youth. We provide a thorough overview of our positionality map, the development of our CAB and our CAB meetings, and the benefits and challenges of incorporating a CAB in neuroscience research.

In order for a just and fair neuroscience that represents the voices from all segments of the population, human neuroscience studies need an interdisciplinary lens that not only includes diverse samples but also takes into account the perspectives and positions of the community members under study. Historically, communities under study, and particularly ethnically and racially minoritized communities, have been left out of the conversations that shape the agenda and direction of neuroscience studies (Mikesell et al., 2013). Even if it’s an unintended consequence, such exclusion promotes the development of biased prevention and intervention approaches, such as medical protocols, mental health recommendations, and governmental policy creation. Our paper outlined how representing the voices of our community partners provide opportunities for bi-directional learning and incorporating their lived experiences to guide approaches for co-created science. As can be seen from our example, the KIND Lab CAB has offered us unique ways for shaping our research by providing substantial feedback for our research design (e.g., inclusion of the questions or topics in our survey modules), recruitment and our outreach (e.g., through the community art workshops we have designed) for our developmental affective neuroscience study on mental health outcomes in preadolescent Latina youth. There is still much work to be explored to reach a true co-created neuroscience; but our team is intentionally working toward expanding this interdisciplinary work into our lab as we continue our dialogue with our CAB members.

Community-engaged research can also have a positive impact on science by increasing public trust in the scientific process. Including communities in the design and interpretation stages of research can serve as a powerful learning opportunity for community members to experience first-hand how research is conducted. This could be especially empowering for young people, given that agency and purpose are central to achieving the developmental tasks of this formative period (Fuligni, 2019). Additionally, including communities in the interpretation and dissemination of the research can also help researchers to identify the aspects of the results that are meaningful to the target populations and—given that communities are rarely the intended audience of scientific publications—provide insights into the alternative ways through which to communicate the study results to those who may be impacted.

Our study also has several limitations. For example, we coincidentally began our CBPR efforts roughly around the same time the COVID-19 pandemic started. Due to the ambiguity and anxiety around this rapidly emerging global pandemic and the new social distancing measures, we carried all our research and community engagement efforts remotely. We held all of our meetings online, which posed challenges to building rapport and trust with the community. However, by remaining in regular contact with our CAB members, maintaining rapport during meetings, and providing food options and compensation for their time, we put effort into creating a trusting meeting environment. As a result, we had full engagement from our CAB members and a willingness to continue participating in future CAB meetings. Some other limitations of our CBPR approach included funding, time, and training constraints. Launching our CBPR approach required that we secured supplementary funding (via an NIH-funded Center Grant from the Center for Health Disparities Research at UCR), spent significant time in revising our methodology, building rapport with community members, and training our research team on best community-engaged research practices. These efforts were substantial and we acknowledge that working within these parameters might pose barriers for other researchers attempting to incorporate CBPR methods in their research.

Finally, we would like to acknowledge the ethical dimensions of CBPR and community-engaged research, particularly power imbalances and inter-cultural sensitivity. Even though CBPR methods aim to empower the community by giving a voice to community members in shaping the research processes or dissemination, in reality a complete leveling of the field is elusive. Power imbalances between researchers and community members– both due to their often more advantaged social locations such as education or social class and institutional affiliation– might create undue influence on the participating community members, shaping CBPR interaction dynamics or outcomes. Similarly, when researchers and the community members are from different social and cultural backgrounds (e.g., in our case, whereas all CAB members were Latina and many of our Research Assistants were Latino/a/x, the majority of our senior research team was not), this can create issues of insufficient inter-cultural sensitivity. The positionality map that we offer in this paper is particularly useful in making these power imbalances and cultural differences visible. Through this exercise, researchers will better be equipped to identify and challenge these ethical issues.

As we outlined in this paper, community-engaged research, and CBPR, is an opportunity to facilitate impactful change via long-term community-academic partnerships in the realm of neuroscience. The mutual cultivation of trust and sharing of cultural and scientific knowledge can bring tangible resources and information to those outside the academic community who are most impacted by the outcomes, propelling toward a just, equitable, and diverse neuroscience.

## Data availability statement

The original contributions presented in this study are included in the article/Supplementary material, further inquiries can be directed to the corresponding author.

## Author contributions

SL, JM, RF, and KM contributed to conception and design of the study and wrote sections of the manuscript. RF provided direction on the community engagement component of the study. SL wrote the first draft of the manuscript. All authors contributed to manuscript revision, read, and approved the submitted version.
