# Supplementary material for: Equity, diversity, and inclusion in developmental neuroscience: Practical lessons from community-based participatory research
Source: Front Integr Neurosci. 2023 Mar 16;16:1007249. doi: 10.3389/fnint.2022.1007249 (PMC10060815; doi:10.3389/fnint.2022.1007249)
Supplement: Supplementary file 1 [file Data_Sheet_1.docx]

Supplementary Material

# 1 CAB Member Agreement

Goals for the Emotional Learning Research Community Advisory Board:

The purpose of the Emotional Learning Research Community Advisory Board (CAB) is to guide and inform the research on emotional learning in youth and adults project led by the researchers in Psychology and Sociology departments at University of California, Riverside. Together we will commit to building an equity-focused research, community capacity and empowerment in conducting research, and developing mutually beneficial relationships between the researchers and the community. The CAB will:

1. Inform research priorities and advise research development based on local needs and culture.
   - We will review and assess existing research protocols used in this study and determine their appropriateness and effectiveness. We will help create future research materials and protocols ensuring the protection of study volunteers and considering community norms and expectations.
2. Advise on study participant recruitment and retention.
   - We will provide input on designing strategies for recruitment of new participants (e.g., identifying new contacts or recruitment sites) and retention of the existing participants of this study.
3. Informing researchers of local issues or concerns.
   - We will inform the research team about information, misinformation, and/or rumors circulating in the community about the study.
4. Assist in the review and analysis of the data collected through this study
   - We will review the findings and provide perspectives from the community.
5. Disseminate study information to local community.
   - We will actively find ways to reach the findings of the study to the other community members and help create dissemination materials or methods (e.g., pamphlets, brochures).

- Attendance at the CAB meetings will be for approximately 60 to 90 minutes during the months of January and June 2020 (date and time T.B.D.).
- Lunch will be provided.
- A $30 stipend, payable to you, will be provided to compensate you for your time at each meeting attended. (**Note that this was modified to $85 for the second meeting with the receipt of additional funding*).
- The first CAB meeting will be held in January 2020 (date and time TBD).

**CAB Community Liaison:** Shayna La Scala, UC Riverside, Department of Sociology, [slas001@ucr.edu](mailto:slas001@ucr.edu)

**UC Riverside Research PI:** Dr. Kalina Michalska, UC Riverside, Department of Psychology, Phone: 951-827-5209, [kalina.michalska@ucr.edu](https://post.ucr.edu/owa/redir.aspx?C=fVk-_pM5sGzNY2ultD4GYDi6uhNkbggfd7et-zWgeqEr6JH1ZuLTCA..&URL=mailto:kalina.michalska@ucr.edu)

**DECLARATION:**

I .................................................................................. certify that I have read and clearly understood the CAB member agreement and do hereby agree to serve as a CAB member in accordance with the above.

Name: .........................................................................................

Address: .......................................................................................

Telephone: ...................................................................................

Email: ...........................................................................................

Signature:........................................... Date: ....................................

*Note: Two copies of this charter should be signed by a CAB member. A copy will be retained by a CAB member while the other will be filed by the CAB liaison.*

# 2 CAB Meeting 1 Agenda

**Agenda**

1. **Intro, 20 min, Shayna welcomes moms and says that we will be doing introductions, starting with Lab director and her collaborator:**

Introductions (research team + CAB members) + ice breaker.

Start with a summary (Kalina and Rengin can share the summary to explain the research briefly: Kalina to focus on brain & mental health outcomes and underrepresentation of Latinx community in existing research, Rengin to focus on the protective effects of ethnic identity, values and socialization).

1. **Consent, 5 min:**

Circulate consent at the meeting for them to read: Shayna to email participants 1:30 hr before the meeting and also screen share during the meeting if they have questions about a certain part of the document . **Do participants have any questions about the consent document?**

1. **Questions to CAB, 40-50 min, moderated by Shayna:**
2. ***Worries/anxieties/research experience:***

- Can you talk about your experiences with our research?
- What do you think your contribution to this research is?
- What things do you like about your participation and what aspects do you not like?
- What was the easiest part of this research for you?
- What was the most challenging part?
- Do you have any worries and anxieties about the research?
- Do you worry about any parts of the consent document (i.e., parts asking questions about sharing data etc.)? If so, what can we do to alleviate the concerns? How can we communicate that the risk is low?
- Are you willing to come in person for a research visit in Fall?

1. ***Dissemination and impact:***

- Are there aspects of this work that you care about? Which parts of our study is meaningful to you and your community?
- Do you talk to other people about your participation in this research? Who do you share your experiences with?
- How can we address your needs the best?
- Are there things that we should emphasize more?
- What information or resources would be useful for you?

1. ***Recruitment:***

- Show them the flyer: what comes to your mind?
- Why do you think some people are not participating? Does it have to do with the incentives, time commitment etc.?
- Is there information that you would want to see out of this study that can motivate your participation?
- How can we recruit from more diverse populations? Or other populations? Are there any forums or groups that we can reach out to?

1. ***Retention:***

- Why are you participating?
- Why do people stop coming?
